# Netrin-G1 regulates fear-like and anxiety-like behaviors in dissociable neural circuits

**DOI:** 10.1038/srep28750

**Published:** 2016-06-27

**Authors:** Qi Zhang, Chie Sano, Akira Masuda, Reiko Ando, Mika Tanaka, Shigeyoshi Itohara

**Affiliations:** 1 Laboratory for Behavioral Genetics, RIKEN Brain Science Institute, Wako, Saitama, 351-0198, Japan

## Abstract

In vertebrate mammals, distributed neural circuits in the brain are involved in emotion-related behavior. Netrin-G1 is a glycosyl-phosphatidylinositol-anchored synaptic adhesion molecule whose deficiency results in impaired fear-like and anxiety-like behaviors under specific circumstances. To understand the cell type and circuit specificity of these responses, we generated netrin-G1 conditional knockout mice with loss of expression in cortical excitatory neurons, inhibitory neurons, or thalamic neurons. Genetic deletion of netrin-G1 in cortical excitatory neurons resulted in altered anxiety-like behavior, but intact fear-like behavior, whereas loss of netrin-G1 in inhibitory neurons resulted in attenuated fear-like behavior, but intact anxiety-like behavior. These data indicate a remarkable double dissociation of fear-like and anxiety-like behaviors involving netrin-G1 in excitatory and inhibitory neurons, respectively. Our findings support a crucial role for netrin-G1 in dissociable neural circuits for the modulation of emotion-related behaviors, and provide genetic models for investigating the mechanisms underlying the dissociation. The results also suggest the involvement of glycosyl-phosphatidylinositol-anchored synaptic adhesion molecules in the development and pathogenesis of emotion-related behavior.

Fear and anxiety are conceptually similar behaviors^1^. Whereas fear is generated by discrete and acute threats, anxiety is evoked by vague and potential dangers^2^. One-third of the human population suffers from fear and/or anxiety disorders at some point in their lifetime^3^, making them the most common psychiatric disorders. Over the last three decades, pharmacology and gene knockout studies revealed the involvement of various genetic factors in the formation of fear and anxiety-related states^4^,^5^. Beyond these molecular characterization studies, recent attention has focused on the use of genetic ablation and optogenetic methods to identify the neural circuits involved in fear-like and anxiety-like behaviors^6^,^7^,^8^,^9^,^10^. Despite such progress, information on genes and circuits alone cannot help us to completely decipher the logic of these diseases, as the same gene could be specifically interconnected with a particular emotional experience through different neural circuits. Therefore, it is important to determine the molecules and neural circuits with key roles in fear and anxiety and to understand how gene-circuit interactions regulate affective behaviors.

Cell adhesion molecules (CAMs) maintain the physical contact between opposing membranes and may be involved in regulating fear and anxiety^11^. Many CAMs are anchored to membranes by covalent linkage to the modified lipid glycosyl-phosphatidylinositol (GPI). Little is known, however, about the circuit basis of GPI adhesion molecules in the modulation of fear and anxiety. Netrin-G1 is an evolutionarily recent member in the family of GPI-anchored CAMs that is specific to vertebrates and localizes mainly on the presynaptic membrane. Netrin-G1 and its paralog netrin-G2 bind with cognate ligands, encode functional synaptic diversity in complementary neural circuits, and confer genetic diversification of mouse behaviors^12^,^13^,^14^,^15^. In addition, netrin-G1 global KO (gKO) mice exhibit marked alterations in fear-like and anxiety-like behaviors under specific circumstance^15^.

To investigate the mechanisms responsible for the emotion-related behavioral deficits observed in netrin-G1 gKO mice, we created mice with Cre-mediated conditional deletions of the netrin-G1 gene in defined cell types of the brain and examined fear-like and anxiety-like behavioral phenotypes. We demonstrated that ablation of netrin-G1 in cortical excitatory neurons selectively alters anxiety-like behavior while ablation of netrin-G1 in inhibitory neurons alters fear-like behavior. Thus, netrin-G1 provides a molecular basis for dissecting the various components involved in regulating emotion-related behaviors.

## Results

### Brain regions are differentially activated by anxiety-like or fear-like behavior in wild-type (WT) and netrin-G1 gKO mice

**Figure 1:**
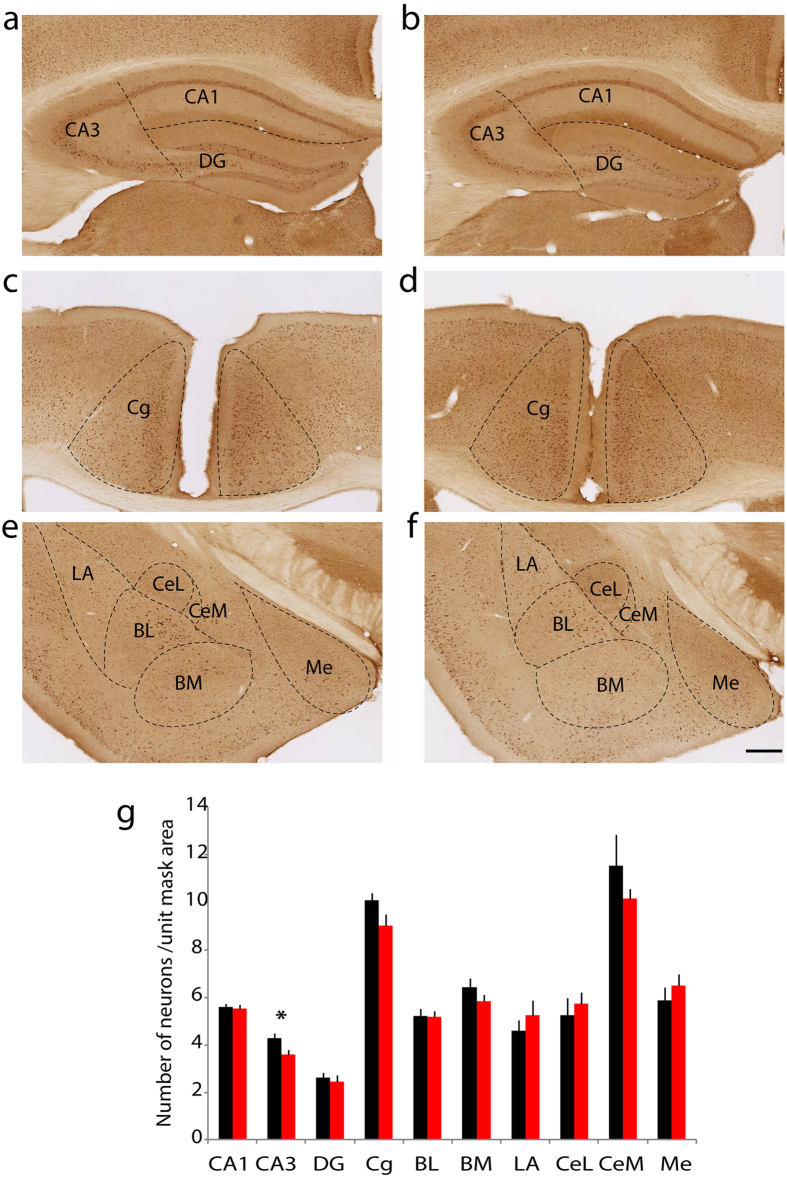
Differential c-Fos expression patterns in netrin-G1 gKO mice after elevated plus maze. (**a**,**c**,**e**) Representative images of hippocampus, cingulate cortex, and amygdala from WT mice. (**b**,**d**,**f**) Representative images of the corresponding brain regions from netrin-G1 gKO mice. (**g**) The number of c-Fos positive cells in each ROI was compared between WT and netrin-G1 gKO mice using Student’s two-tailed t test, n = 8, (2 animals × 4 sections) per genotype. Netrin-G1 gKO mice had significantly fewer c-Fos positive cells in CA3 (p = 0.01) and marginally fewer c-Fos positive cells in the cingulate cortex (Cg, p = 0.066). The number of c-Fos positive cells was not significantly different in the CA1 (p = 0.84), dentate gyrus (DG, p = 0.68), or in any of the amygdala subnuclei: basolateral (BL, p = 0.95), basomedial (BM, p = 0.24), lateral (LA, p = 0.39), lateral central nucleus (CeL, p = 0.58), medial central nucleus (CeM, p = 0.33), and medial nucleus (Me, p = 0.44). Black columns represent WT and red columns represent gKO, and the data are represented as mean + SEM. *p < 0.05; scale bar: 500 μm (**a**,**b**); 250 μm (**c**,**d**); 280 μm (**e**,**f**).

**Figure 2 f2:**
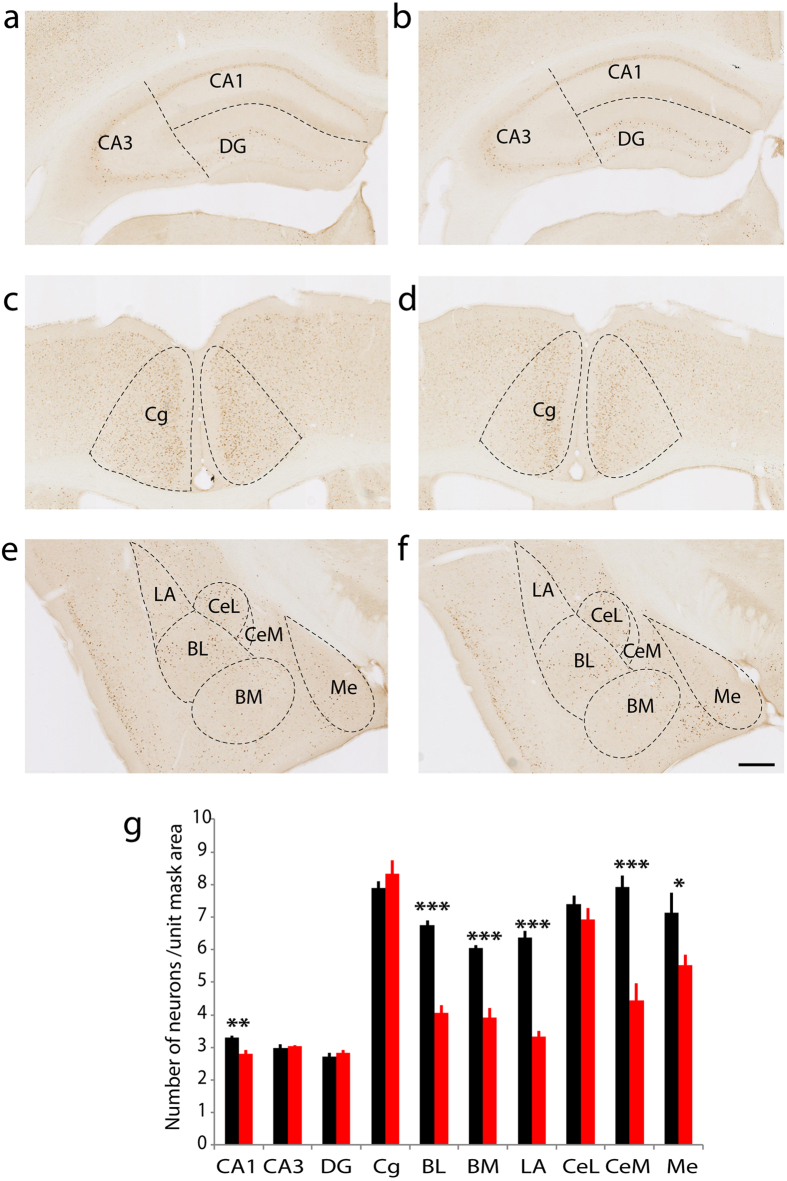
Differential c-Fos expression patterns in netrin-G1 gKO mice after fear conditioning. (**a**,**c**,**e**) Representative images of hippocampus, cingulate cortex, and amygdala from WT mice. (**b**,**d**,**f**) Representative images of the corresponding brain regions from netrin-G1 gKO. (**g**) The number of c-Fos positive cells in each ROI was compared between WT and netrin-G1 gKO mice using Student’s two-tailed t test, n = 8, (4 animals × 2 sections) per genotype. Netrin-G1 gKO mice had significantly fewer c-Fos-positive cells in CA1 compared with WT mice (p < 0.01), while there was no significant difference in the CA3 (p = 0.62), DG (p = 0.44) and Cg (p = 0.31). In the amygdala, the number of c-Fos positive cells was markedly decreased in almost all subnuclei of the amygdala, including the BL (p < 0.001), BM (p < 0.001), LA (p < 0.001), CeM (p < 0.001), and Me (p = 0.03). In the CeL, however, there was no significant difference between genotypes (p = 0.31). Black columns represent WT and red columns represent gKO, and the data are represented as the mean + SEM. *p < 0.05, **p < 0.01, ***p < 0.001; scale bar: 500 μm (**a**,**b**); 250 μm (**c**,**d**); 280 μm (**e**,**f**).

Our previous study demonstrated that in an EPM, WT mice display a stronger place preference for the closed arms over the open arms, while netrin-G1 gKO mice exhibit no preference. However, netrin-G1 gKO mice spent an equivalent time as WT mice at the center of an open field box, indicating reduced anxiety-like behavior in a specific context^15^. In a fear conditioning test (FC), the freezing response is significantly reduced in netrin-G1 gKO mice during the conditioning stage as well as in the context-dependent and cue-dependent memory testing stages, indicating deficits in fear-like behavior^15^. To gain insight into the neuronal circuits underlying these behavioral abnormalities of netrin-G1 gKO mice, we examined c-Fos positive neurons in WT and netrin-G1 gKO mice exposed to either the EPM or the acquisition phase of the FC test. The behavioral phenotypes of the mice analyzed were consistent with those in the previous study^15^ (Supplementary Figs 1 and 2). Differential c-Fos expression patterns were observed between WT and netrin-G1 gKO mice in various brain regions, including the anterior cingulate cortex, hippocampus, and amygdala. In the EPM group (Fig. 1), netrin-G1 gKO mice had a significantly lower number of c-Fos-positive cells in the CA3 region of the hippocampus (WT: 4.3 ± 0.2, gKO: 3.6 ± 0.2) and a marginally lower number in the cingulate cortex (WT: 10.1 ± 0.3, gKO: 9.0 ± 0.5) compared with WT mice. No differences were observed between WT and netrin-G1 mice in the CA1, dentate gyrus (DG), or any amygdala subnuclei. In the FC group (Fig. 2), netrin-G1 gKO mice had significantly fewer c-Fos positive cells in the CA1 region than WT mice (WT: 3.3 ± 0.06, gKO: 2.8 ± 0.1), but the number of c-Fos positive cells in the CA3 and DG did not differ between genotypes. The number of c-Fos positive cells in the cingulate cortex (Cg) was not different between genotype either. Netrin-G1 gKO mice had strikingly fewer c-Fos positive cells in almost all the amygdala subnuclei, including the lateral (WT: 6.4 ± 0.2, gKO: 3.3 ± 0.2), basolateral (WT: 6.8 ± 0.1, gKO: 4.1 ± 0.2), basomedial nuclei (WT: 6.1 ± 0.1, gKO: 3.9 ± 0.3), medial sector of the central nucleus (WT: 7.9 ± 0.4, gKO: 4.4 ± 0.5), and the medial nucleus (WT: 7.1 ± 0.6, gKO: 5.5 ± 0.3), but not in the lateral sector of the central nucleus. The c-Fos expression patterns did not differ between genotypes in the home-cage controls (Supplementary Fig. 3). These findings indicate that ablation of netrin-G1 alters the neuronal response of different brain regions during EPM and FC, and suggest that netrin-G1 regulates fear-like and anxiety-like behavior by mechanisms involving different neural circuits.

### Generation of NetrinG1^f/f^ mice

**Figure 3 f3:**
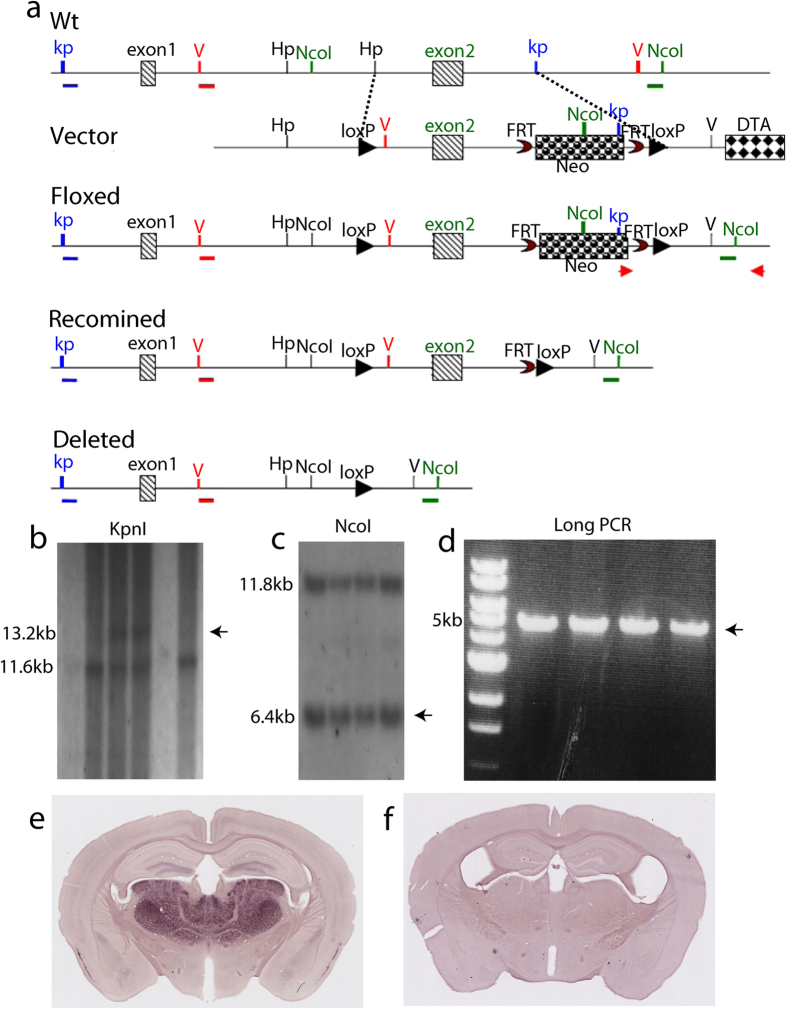
Construction and validation of netrin-G1 floxed mice. (**a**) Schematic diagram of the strategy to generate netrin-G1 floxed mice. The targeting vector contains exon 2 in the targeting region. The 5′ probe with *KpnI* digest and 3′ probe with *NcoI* digest are indicated. The PCR primers for the targeted alleles are shown (red arrows). (**b**) Targeted events were identified by Southern blot analysis of *KpnI*-digested genomic ES cell DNA with a 5′-flanking probe, the WT allele 11.5 kb and the targeted allele 13.2 kb. (**c**) Southern blot analysis of *NcoI*-digested genomic ES cell DNA with a 3′-flanking probe, the WT allele 11.8 kb, and the targeted allele 6.4 kb. (**d**) The 3′ targeting was further confirmed by long PCR, which produced a 5-kb fragment. (**e**,**f**) ISH of NetrinG1^f/f^ and NetrinG1^f/f^: CAG-Cre^+^, respectively, revealed that netrin-G1 expression was totally ablated in NetrinG1^f/f^: CAG-Cre^+^ mice. Scale bar: 1 mm.

To precisely localize the neuronal populations responsible for the decreased fear and anxiety-like behavior in netrin-G1 gKO mice, we generated mice with a conditional knockout (cKO) of the netrin-G1 gene using the Cre-loxP system. The floxed netrin-G1 allele was generated by a gene-targeting strategy using C57BL/6-derived MS12 embryonic stem (ES) cells^16^, as shown in Fig. 3a. Exon 2 encoding the signal peptides and 25% of domain VI was surrounded by a pair of loxP sequences. The successfully targeted ES cells were confirmed by Southern blot analysis and long polymerase chain reaction (PCR; Fig. 3b–d). The germ-line transmittants of the targeted ES clone were crossed with transgenic CAG-Flpe mice^16^ to excise the Neo cassette to obtain the NetrinG1^f/+^mice. The resulting heterozygous offspring were further crossbred with C57BL/6J, and interbred to obtain homozygous NetrinG1^f/f^ mice. To confirm the functionality of the loxP sites *in vivo*, we crossed these mice with CAG-Cre mice^17^ in which Cre recombinase is constitutively and broadly expressed by the ubiquitous CAG promoter, and obtained NetrinG1^−/−^:Cre^+^ mice. *In situ* hybridization (ISH) revealed the characteristic expression patterns of the netrin-G1 gene in NetrinG1^f/f^ mice and the lack of netrin-G1 signals in the NetrinG1^−/−^:Cre^+^ mice (Fig. 3e,f). Moreover, NetrinG1^−/−^:Cre^+^, but not NetrinG1^f/f^ mice, had a smaller body size, similar to netrin-G1 gKO mice^12^. These results revealed the functional integrity of the floxed allele and the competency of the loxP sequences.

### Ablation of netrin-G1 in cortical excitatory neurons decreased anxiety-like behavior in the EPM

**Figure 4:**
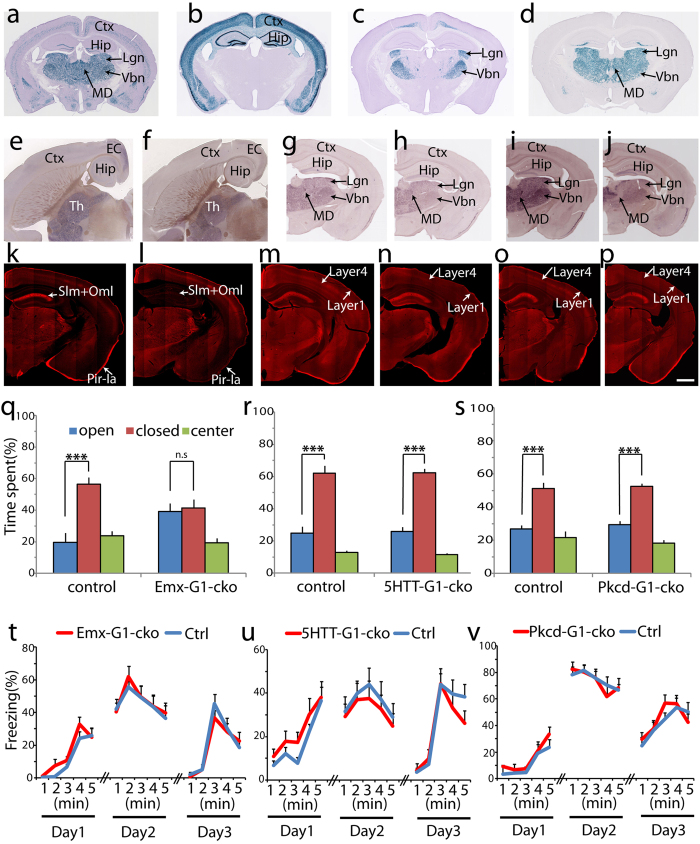
Ablation of netrin-G1 in excitatory neurons in the cortex and thalamus. (**a**) X-gal staining of coronal sections of a NetrinG1-NLS-LacZ mouse. (**b–d**) X-gal staining of Rosa-NLS-LacZ reporter mice crossed with Emx1-Cre (**b**), 5HTT-Cre (**c**), and Pkcd-Cre (**d**) lines, respectively. (**e**–**j**) Representative images from ISH of netrin-G1 (Ntng1). Horizontal sections from control (**e**) and Emx1-G1-cKO mice (**f**) show that cortical expression of netrin-G1 was abolished. Coronal sections from control (**g**) and 5HTT-G1-cKO (**h**) mice show the selective reduction of netrin-G1 in the ventral basal nucleus (Vbn) and lateral geniculate nucleus (Lgn). Coronal sections from control (**i**) and Pkcd-G1-cKO (**j**) mice show the dramatic reduction of netrin-G1 in almost the entire thalamus. (**k**–**p**) Representative images from immunohistochemistry of netrin-G1. In Emx-G1-cKO (l) mice, netrin-G1 disappeared in the Slm and Oml of the hippocampus and piriform cortex layer I compared with the control (**k**). In 5HTT-G1-cKO (**n**) mice, netrin-G1 dramatically decreased in cortex layer IV compared with the control (**m**). In Pkcd-G1-cKO (**p**) mice, netrin-G1 disappeared from cortical layers IV and I compared with the control (**o**). Scale bar: 1 mm (**a**–**f**); 800 μm (**g**–**j**); 750 μm (**k**–**p**). (**q**–**s**) Behaviors in the EPM. (**q**) The littermate control mice spent a significantly greater amount of time in the closed arms while Emx1-G1-cKO mice showed no preference between open and closed arms (n = 11/genotype). (**r**) 5HTT-G1-cKO mice showed no differences from the control mice (n = 12 per genotype). (**s**) Pkcd-G1-cKO mice showed no differences from the control mice (n = 12 per genotype). Shown are the mean + SEM. Unpaired two-tailed Student’s t test; ***p < 0.001; ns, not significant. (**t**–**v**) Percent freezing behavior in the fear conditioning (day 1), context-dependent memory (day 2), and cue-dependent memory (day 3) tests. (**t**) No significant effect of genotype was detected in Emx-G1-cKO mice in all tests (n = 14 per genotype). (**u**) No significant effect of genotype was detected in 5HTT-G1-cKO mice in all tests (n = 12 per genotype). (**v**) No significant effect of genotype was detected in Pkcd-G1-cKO mice in all tests (n = 12 per genotype). Shown are the mean + SEM. Two-way ANOVA was conducted between time and genotype.

Netrin-G1 is mainly expressed throughout the dorsal thalamus and specific layers of the cortex^18^ (Fig. 4a). To dissect these representative circuits, we first crossed NetrinG1^f/f^ mice with the Emx1-Cre line, which induces recombination in excitatory neurons in the cerebral cortex and limbic structures (Fig. 4b)^19^, and with the 5HTT-Cre line^20^, which induces recombination in excitatory neurons in the first-order thalamic relay nuclei (Fig. 4c) and raphe nuclei. Both Emx1-G1-cKO and 5HTT-G1-cKO mice were born and developed normally, and the body size did not differ from that of the WT control mice. Immunohistochemistry and ISH clearly showed a loss of netrin-G1 expression in these mice in the expected brain areas. In Emx1-G1-cKO mice, netrin-G1 mRNA expression was drastically lower in the cortex, but unaltered in the thalamus (Fig. 4e,f). Netrin-G1 proteins were almost completely abolished in the hippocampus stratum lacunosum-molecular (Slm) and outer molecular layer (Oml) (Fig. 4k,l). In WT mice, netrin-G1 protein distributes on lateral perforant path axons originating from lateral entorhinal cortex layer 2 neurons and temporoammonic path axons originating from entorhinal cortex layer 3 neurons^12^. In the Emx1-G1-cKO mice, the immune signals were also dramatically decreased in layer 1a of the piriform cortex (Fig. 4k,l), representing the terminal layer of mitral cell axons from the olfactory bulb^21^. In 5HTT-G1-cKO mice, netrin-G1 mRNA was well maintained in the cortex, but significantly decreased in the ventral basal (Vbn), lateral geniculate (Lgn), and medial geniculate nuclei (Mgn) of the thalamus (Fig. 4g,h). Immunohistochemistry revealed a marked decrease in netrin-G1 in layer 4 of the cortex (Fig. 4m,n), representing core-type thalamocortical axons from the first-order relay nuclei of the thalamus, including the ventral basal, lateral geniculate, and medial geniculate nuclei^22^. The weak signals in layer 4 may come from the ventral lateral nucleus or higher-order relay nuclei^22^,^23^. The signals in layer 1, representing matrix-type thalamocortical axons originating from non-specific thalamic nuclei^22^, were highly conserved (Fig. 4m,n).

We investigated the effects of conditional ablation of netrin-G1 on performance in the EPM and FC test. The control mouse groups avoided entering the open arms of the EPM and spent significantly more time in the closed arms (open arms: 19.7 ± 6.0%; closed arms: 56.4 ± 4.5%). Emx1-G1-cKO mice, however, exhibited no preference for the closed arms (open arms: 39.3 ± 5.0%; closed arms: 41.3 ± 5.6%; Fig. 4q), indicating less anxiety-like behavior in the EPM, similar to netrin-G1 gKO mice. 5HTT-G1-cKO mice spent significantly more time in the closed arms than in the open arms (open arms: 26.0 ± 2.8%; closed arms: 62.3 ± 2.6%), similar to their control group (open arms: 24.9 ± 4.1%; closed arms: 62.1 ± 4.6%; Fig. 4r). Locomotor activity was not altered in either the Emx1-G1-cKO or 5HTT-G1-cKO mice (Supplementary Fig. 4). These findings indicate that netrin-G1 in forebrain excitatory neurons, but not in the thalamic first-order relay nuclei, has a key role in regulating anxiety-like behaviors in the EPM. In the FC test, however, both Emx1-G1-cKO and 5HTT-G1-cKO mice exhibited comparable freezing responses with their own control group at all stages, including the conditioning stage, the contextual memory test, and the cued memory test (Fig. 4t,u; there is no interaction between the effects of genotype and time on freezing for both groups: Emx1-G1-cKO versus control, F(4,130) = 0.68, p = 0.60 on day 1; F(4,130) = 0.11, p = 0.98 on day 2; F(4,130) = 0.57, p = 0.69 on day3; 5HTT-G1-cKO versus control, F(4,110) = 0.20, p = 0.94 on day 1; F(4,110) = 0.03, p = 0.99 on day 2; F(4,110) = 0.71, p = 0.59 on day3; and no effect of genotype on freezing: Emx1-G1-cKO versus control, F(1,130) = 3.05, p = 0.08 on day 1; F(1,130) = 0.16, p = 0.69 on day 2; and F(1,130) = 0.38, p = 0.54 on day 3; 5HTT-G1-cKO versus control, F(1,110) = 3.36, p = 0.07 on day 1; F(1,110) = 0.83, p = 0.37 on day 2; and F(1,110) = 0.83, p = 0.37 on day 3). These results indicate that the cells targeted in the Emx1-G1-cKO and 5HTT-G1-cKO mice do not mediate fear-like behavior. This was unexpected as the first-order relay nuclei are key brain regions involved in fear^24^,^25^, and netrin-G1 is strongly expressed in these nuclei.

We then hypothesized that netrin-G1 in other parts of the thalamus has an important role in regulating fear-like behavior. To test this hypothesis, we generated a novel Cre transgenic mouse line using the *Pkcd* promoter. The selected Pkcd-Cre line induced recombination in almost the entire dorsal thalamus except for the paraventricular thalamic nucleus and the central part of the mediodorsal thalamic nucleus (Fig. 4d). We crossed NetrinG1^f/f^ mice with Pkcd-Cre mice to obtain Pkcd-G1-cKO mice. Compared with control mice, netrin-G1 ISH signals in Pkcd-G1-cKO mice were dramatically lower throughout almost the entire thalamus (Fig. 4i,j). Immunohistochemistry revealed almost a complete disappearance of the signals in layer 4 and very weak signals in layer 1 of the cortex, compared with the control (Fig. 4o,p; arrows). This observation indicates that much broader and more efficient Cre recombination was induced in the thalamus by crossing with the Pkcd-Cre line than the 5HTT-Cre line. The responses of the Pkcd-G1-cKO mice (open arms: 29.4 ± 2.3%; closed arms: 52.5 ± 1.9%) did not differ from those of control mice (open arms: 27.0 ± 2.0%; closed arms: 51.3 ± 3.4%) in the EPM (Fig. 4s). The Pkcd-G1-cKO group was not significantly different from its control group in any of the phases of the FC test (Fig. 4v; there is no interaction between the effects of genotype and time on freezing: F(4,95) = 0.38, p = 0.82 on day 1; F(4,95) = 0.27, p = 0.90 on day 2; F(4,95) = 0.62, p = 0.65 on day 3; and no effect of genotype on freezing: F(1,95) = 3.33, p = 0.07 on day 1; F(1,95) = 0.03, p = 0.87 on day 2; and F(1,95) = 0.39, p = 0.54 on day 3). These findings suggest that netrin-G1 does not regulate fear-like or anxiety-like behavior through the thalamus, despite its very strong expression in this brain structure.

### Expression of netrin-G1 in inhibitory neurons

To date, all previous studies of netrin-Gs and their binding partners, netrin-G ligands (NGLs), have focused on excitatory neurons^12^,^13^,^26^,^27^. To clearly identify netrin-G1 positive cells, we systematically analyzed netrin-G1 expression in inhibitory neurons using heterozygous netrin-G1 gKO mice (NetrinG1-NLS-LacZ-knockin mice)^12^. In this mouse line, a Lac-Z reporter gene with a nuclear localization signal is inserted in-frame after the initiation codon of netrin-G1, which provides a highly sensitive method for detecting the pattern of netrin-G1 expression at a single cell resolution level by X-gal staining (Fig. 4a) or ß-gal immunohistochemistry.

We first confirmed the neuronal identity of netrin-G1 positive cells by double immunostaining coronal sections of adult mouse brain with anti-NeuN and anti-ß-galactosidase antibodies (Supplementary Fig. 5). The NeuN signal was detected in all the ß-gal-positive neurons across all brain regions tested, including the cortex (Supplementary Fig. 5a,b), hippocampus (Supplementary Fig. 5c,d), zona incerta (ZI; Supplementary Fig. 5e,f), thalamus (Supplementary Fig. 5g–i), and amygdala (Supplementary Fig. 5j–l). Consistent with the results of the X-gal staining (Fig. 4a), netrin-G1 was not expressed in any neurons in the reticular nucleus (Supplementary Fig. 5i).

**Figure 5:**
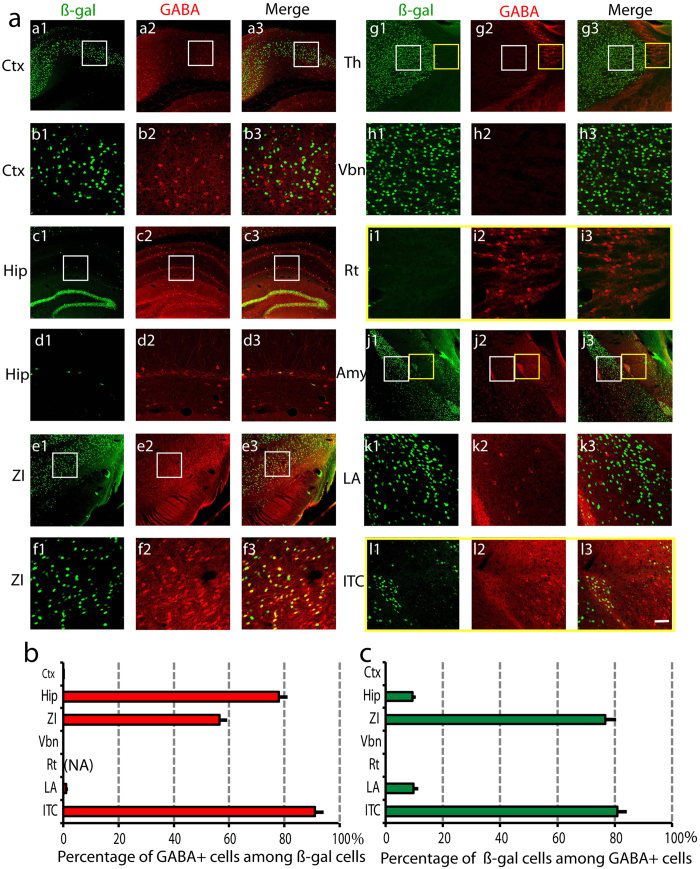
Netrin-G1 expression in inhibitory neurons. (**a**) Coronal sections containing different brain regions were co-immunostained with antibodies against ß-gal (1: green) and GABA (2: red), and colocalization was observed (3: green + red). (a1–a3) represent cortex (Ctx) and (b1–b3) are higher magnified images of the areas indicated by the white boxes in a1–a3. (c1–c3) represent hippocampus (Hip) and (d1–d3) are higher magnified images (white boxes in c1–c3). (e1–e3) represent zona incerta (ZI) and (f1–f3) are higher magnified images (white boxes in e1–e3). (g1–g3) represent thalamus and (h1–h3) and (i1–i3) are higher magnified images from Vbn (white boxes in g1–g3) and reticular nucleus (Rt; yellow boxes in g1–g3). (j1–j3) represent amygdala, (k1–k3) and (l1–l3) are higher magnified images from LA (white boxes in j1–j3) and the dorsal intercalated cluster (ITC; yellow boxes in j1–j3). Scale bar: 120 μm (**a**,**c**,**e**,**g**,**j**); 30 μm (**b**,**d**,**f**,**h**,**i**,**k**,**l**). (**b**) Mean percentage of GABA-positive cells among ß-gal-positive cells for each ROI was calculated (n = 16, 4 animals × 4 sections). NA represents “not applicable”, because there is no ß-gal positive cell in Rt and 0 cannot be a denominator. (**c**) Mean percentage of ß-gal-positive cells among GABA-positive cells for each ROI was calculated (n = 16, 4 animals × 4 sections). We analyzed a total of 3340 (Ctx), 924 (Hip), 1779 (ZI), 6972 (Tha), 5228 (LA), and 976 (ITC) cells.

We then applied double immunostaining with anti-GABA and anti-ß-galactosidase antibodies (Fig. 5). In the cortex, there was no overlap of ß-gal and GABA signals, indicating that netrin-G1 is only expressed in cortical excitatory neurons (Fig. 5a(a1–b3, [Fig f2], [Fig f3]),b,c). In the thalamus, there was also no overlap of the two signals (Fig. 5a(g1–h3),b,c). The thalamic reticular nucleus is the main GABAergic structure in the thalamus. Consistent with the X-gal staining (Fig. 4a), no ß-gal-positive neurons were detected in this area (Fig. 5a(g1–g3,i1–i3),b,c). In the hippocampus, netrin-G1 was expressed in sparsely distributed cells and 78.0 ± 2.9% of them were GABA-positive (Fig. 5a(c1–d3),b). This indicates that, except for granule cells, netrin-G1 is also expressed in inhibitory neurons in the hippocampus, representing 9.4 ± 1.1% of the hippocampal GABA-positive neurons (Fig. 5a(c1–d3), c). In the amygdala, netrin-G1 was mainly detected in the lateral nucleus (LA), and only 1.1 ± 0.5% of these neurons were GABA-positive (Fig. 5a(j1–k3),b). This small population comprised 9.7 ± 1.7% of the LA GABAergic neurons (Fig. 5c). In the intercalated clusters, 91.0 ± 3.0% of the ß-gal-positive neurons were also GABA-positive and 80.9 ± 3.3% of the GABA-positive neurons expressed ß-gal (Fig. 5a(j1–l3),b,c). Neurons positive for ß-gal were also scattered in other amygdala subnuclei, including the basolateral, basomedial, and medial nuclei, which were mainly GABA-negative (Fig. 5a(j1–j3)). In the ZI, 56.5 ± 2.6% of the ß-gal-positive neurons were GABA-positive, and 76.6 ± 3.9% of the GABA-positive neurons expressed ß-gal (Fig. 5a(e1–f3),b,c). Therefore, netrin-G1 was expressed in some populations of inhibitory neurons of the hippocampus, amygdala, and ZI.

### Ablation of netrin-G1 in inhibitory neurons impaired fear-like behavior in the FC test

**Figure 6:**
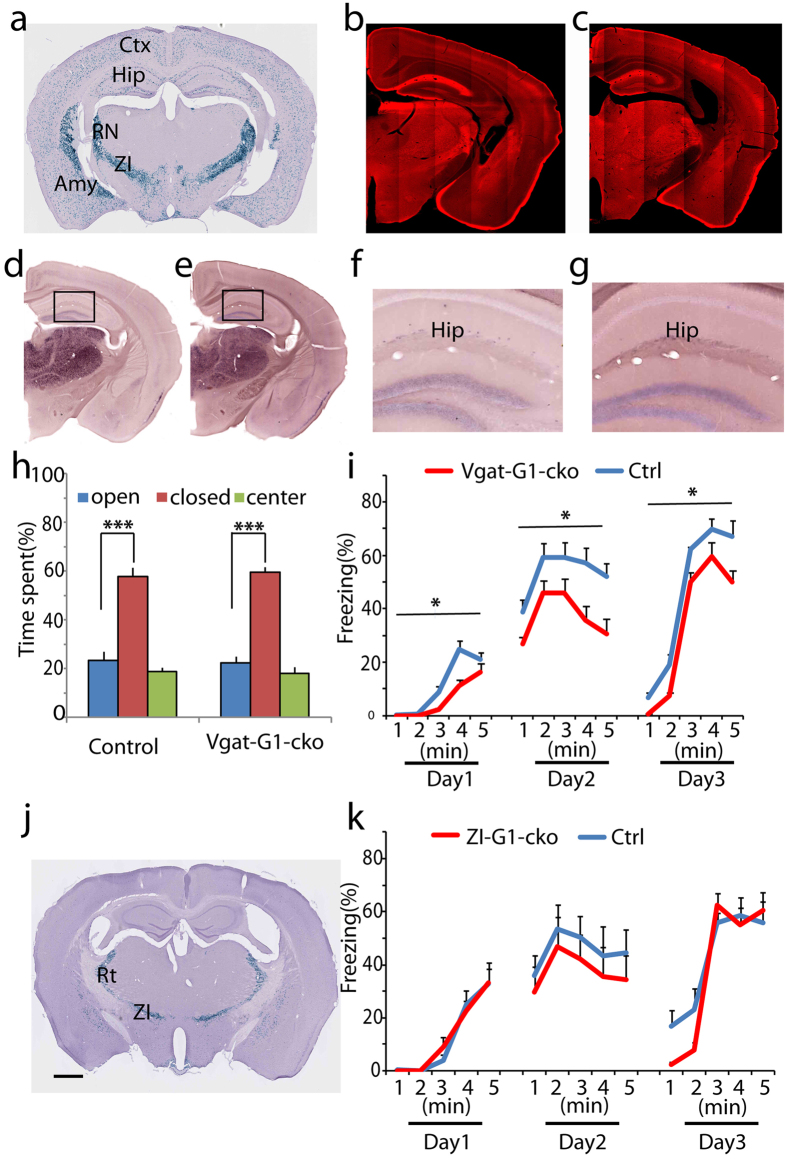
Ablation of netrin-G1 in inhibitory neurons. (**a**) X-gal staining of coronal sections of Rosa-NLS-LacZ reporter mice crossed with the Vgat-Cre line. (**b**,**c**) Representative immunohistochemical images of netrin-G1 in control and Vgat-G1-cKO mice, respectively. No apparent difference was observed. (**d**–**g**) Representative images of ISH of netrin-G1 from the control (**d**,**f**) and Vgat-G1-cKO (**e**,**g**) mice, respectively. (**f**,**g**) are magnified images of the areas marked with a square in (**d**,**e**), respectively. Note that in (**f**) the positive signal is sparsely distributed, and the signal is dramatically decreased in (**g**). Scale bar: 1 mm (**a**,**d**,**e**,**j**); 750 μm (**b**,**c**); 200 μm (**f**,**g**). (**h**) Percent time spent in the open and closed arms of the EPM. Both the control and Vgat-G1-cKO mice spent significantly greater amounts of time in the closed arms than in the open arms. Statistical analysis between percent time spent in the open arms and the closed arms was conducted using the unpaired two-tailed Student’s t test (n = 9 per genotype). Columns and bars represent the mean + SEM, respectively. ***p < 0.001. (**i**) Percent time exhibiting freezing behavior during fear conditioning (day1), context-dependent memory (day2) and cue-dependent memory (day3). Mean freezing duration was significantly reduced in Vgat-G1-cKO mice during all 3 stages. *p < 0.05 (n = 9 per genotype). Two-way ANOVA was conducted between time and genotype. Colored lines and bars represent the mean + SEM, respectively. (**j**) X-gal staining of coronal sections of Rosa-NLS-LacZ reporter mice crossed with the ZI-Cre line showing that Cre was mainly expressed in the reticular nucleus and zona incerta, with sparse expression in the striatum. (**k**) In the FC test, no significant effect of genotype on freezing behavior was detected between ZI-G1-cKO and control groups during the conditioning, and contextual and cued fear-related memory retrieval phases. Two-way ANOVA was conducted between time and genotype (n = 9 per genotype). Colored lines and bars represent the mean + SEM, respectively.

To examine if netrin-G1 on inhibitory neurons is involved in regulating emotion-related behaviors, we crossed netrin-G1^f/f^ mice with the Vgat-Cre line, which induces recombination in all inhibitory neurons throughout the brain (Fig. 6a)^28^. Gross immunohistochemical signals, largely representing netrin-G1 on excitatory neurons, were comparable between the Vgat-G1-cKO and control mice (Fig. 6b,c). ISH signals from scattered inhibitory neurons in the hippocampus, however, dramatically decreased in Vgat-G1-cKO mice, revealing successful knockout of netrin-G1 in most inhibitory neurons (Fig. 6d–g).

In the EPM test, Vgat-G1-cKO mice (open arms: 22.3 ± 2.7%; closed arms: 59.5 ± 2.1%) exhibited a similar level of anxiety-like behavior as control mice (open arms: 23.5 ± 3.6%; closed arms: 57.7 ± 3.7%; Fig. 6h). No differences in locomotor activity were detected (Supplementary Fig. 4). Vgat-G1-cKO mice, however, had significantly lower freezing levels during the conditioning stage and subsequent context and cued fear memory test stages (Fig. 6i; there is no interaction between the effects of genotype and time on freezing: F(4,80) = 0.92, p = 0.46 on day 1; F(4,80) = 0.11, p = 0.98 on day 2; F(4,80) = 0.15, p = 0.96 on day3; but an effect of genotype on freezing: F(1,80) = 4.18, p = 0.04 on day 1; F(1,80) = 6.60, p = 0.012 on day 2; and F(1,80) = 6.14, p = 0.015 on day 3). These findings revealed the important role of netrin-G1 in the inhibitory neuronal network to specifically regulate fear-like behavior, but not anxiety-like behavior. The ZI is one of the main brain regions containing netrin-G1 positive inhibitory neurons. Thus, we used a novel Vgat-Cre (ZI-Cre) subline that selectively induces recombination in the reticular thalamic nucleus and ZI (Fig. 6j). We observed no difference at any of the stages in FC test between ZI-G1 cKO and control mice (Fig. 6k; there is no interaction between the effects of genotype and time on freezing: F(4,80) = 0.27, p = 0.90 on day 1; F(4,80) = 0.013, p = 1.00 on day 2; F(4,80) = 1.49, p = 0.21 on day3; and no effect of genotype on freezing: F(1,80) = 0.035, p = 0.85 on day 1; F(1,80) = 1.68 p = 0.20 on day 2; and F(1,80) = 1.43 p = 0.24 on day 3). These results suggest that netrin-G1 in inhibitory neurons, excluding ZI cells, attenuates fear-like behavior in the FC test.

## Discussion

Whether anxiety and fear are distinct or indistinguishable states has long been debated. Many early studies revealed that the brain areas, neurotransmitters, peptides, hormones, and neuromodulators involved in fear and anxiety largely overlap. In particular, fear- and anxiety-like behaviors mostly share similar output circuits. The application of recently developed circuit-centered approaches allows researchers to investigate the divergence and convergence of fear-related and anxiety-related circuits at high spatial and temporal resolution^29^. However, many questions remain unclear as to the molecular basis for dissecting the various components involved in regulating fear and anxiety. The GPI-anchored synaptic adhesion molecule netrin-G1 is highly expressed in many brain regions associated with regulating emotion. In this study, we demonstrated that anxiety-like behavior in the EPM and the conditioning stage of FC test triggered differential induction patterns of an immediate-early gene in netrin-G1 gKO mice. In addition, we investigated the effect of conditional mutagenesis of netrin-G1 on adaptive behaviors mediated by excitatory neurons in the cortex and limbic areas, thalamic neurons, whole brain inhibitory neurons, and ZI inhibitory neurons. The behavioral phenotypes of cKO mice demonstrated that fear- and anxiety-like behavior could be genetically mapped through netrin-G1 onto two opposing networks – excitatory and inhibitory networks. Our observations support the concept that fear- and anxiety-like behaviors are distinguishable as different entities. Most importantly, the findings of the present study establish for the first time that a single GPI-anchored molecule can have multiple overlapping roles in the diversification of adaptive behavior depending on its expression pattern and function in specific cell types or neural circuits.

GABA and glycine have well-established roles in orchestrating the circuit changes in the regulation of fear^9^,^30^,^31^,^32^,^33^. Previous studies examining the role of netrin-Gs have primarily focused on the function of excitatory neurons^13^,^26^,^27^,^34^, whilst this present study also revealed the expression and behavioral function of netrin-G1 in inhibitory neurons. Many GPI adhesion molecules, including contactin^35^, semaphorinA^36^, ephrinA^37^, NCAM-120^38^, kilon, lysosomal-associated membrane protein (LAMP), and opioid-binding cell adhesion molecule^39^,^40^,^41^,^42^, localize at the synapse. However thus far, only the effects of three GPI-anchored synaptic adhesion molecules on fear and anxiety have been investigated, and they are NCAM, ephrin-A2/-A3 and LAMP^42^,^43^,^44^,^45^,^46^,^47^,^48^,^49^. Some studies showed that NCAM has an effect on affective behavior^44^,^45^,^46^,^47^. However, it is difficult to dissociate the effects of GPI-anchored NCAM120 from those of the other isoforms (NCAM180 and 140, which are both transmembrane proteins with large intracellular domains) because mutant mice used in these studies^44^,^45^,^46^,^47^ lacked all three isoforms. Wurzman and colleagues demonstrated that ephrin-A2/-A3 double-knockout mice exhibit increased anxiety^48^, while LAMP KO mice was reported to exhibit reduced anxiety^42^,^43^,^49^. It is notable that NCAM, ephrinA, and LAMP are also involved in altered GABAergic functions^37^,^49^,^50^. Some studies demonstrate that deletion of transmembrane adhesion molecules alters inhibitory synapse function and is associated with an emotional phenotype^51^. However, at least to our knowledge, no studies have examined whether the altered GABAergic functions are related to the regulation of fear- or anxiety-like behaviors in the mutated mice of GPI-anchored adhesion molecules. Our investigation of netrin-G1 gKO and cKO mice provides new evidence for the important role of GPI-anchored synaptic adhesion molecules in modulating emotion-related behavior, suggesting that there may be a general rule in which GPI-anchored molecules modify certain behavioral states by adjusting inhibitory functions. It is noteworthy that netrin-G1-postive GABAergic cells are enriched in intercalated clusters and the internal capsule of the amygdala, which will provide a powerful genetic tool to precisely target these cell populations and to explore the effects of local inhibition and disinhibition on different emotional states.

While this study has revealed new insights into the role of netrin-G1, many questions are yet to be resolved. One limitation of the present study is that the Cre lines used were not specific to a single neural circuit. The Emx1-G1-cKO and Vgat-G1-cKO phenotypes therefore may result from the sum of all the distributed and highly interconnected neural circuits within the cortical excitatory and inhibitory networks. However, we cannot exclude the possibility that a selective circuit plays a key role. The c-Fos staining showed that after the EPM, brain activity in the CA3 was significantly lower in the netrin-G1 gKO mice than in the WT mice. As netrin-G1 is not expressed in CA3 cells, and CA3 mainly receives inputs from the entorhinal cortex and DG where netrin-G1 is strongly expressed, it is possible that the netrin-G1-positive cells in the entorhinal cortex and/or DG are mainly responsible for regulating anxiety-like behavior. On the other hand, after the training phase of the FC test, neuronal activity was significantly lower in the CA1 and several subnuclei of the amygdala in netrin-G1 gKO mice. Netrin-G1 is expressed in a subpopulation of inhibitory neurons in the hippocampus and inhibitory neurons of the intercalated clusters in the amygdala. The connections between the hippocampus and BLA are involved in fear and anxiety-like behaviors^52^,^53^,^54^,^55^. Therefore it is conceivable that the local circuits within the amygdala or hippocampus and/or the interplay between these two regions are responsible for the netrin-G1 mediated fear regulation. Further virus-mediated cell-specific genetic studies (including local deletion and rescue experiments) may help to identify the precise neural circuits through which netrin-G1 regulates fear and anxiety-like behavior. Besides, netrin-G1 laterally interacts with the tyrosine phosphatase leukocyte antigen-related receptors^56^ and trans-synaptically interacts with NGL-1 in excitatory neurons^14^. Whether the same interactions and signaling pathways exist for netrin-G1-positive inhibitory neurons requires further investigation. Our previous study on acute hippocampus slices revealed that netrin-G1 gKO mice have attenuated short and long-term synaptic plasticity at specific neural circuits^13^. Further studies are needed to address the dynamics of neuronal plasticity in different brain regions of behavioral netrin-G1 cKO mice. Netrin-G1 is suggested to be involved in schizophrenia and bipolar disorder^57^. Our study sheds light on the mechanisms underlying the emotional deficits of these mental disorders.

## Materials and Methods

All experimental protocols were approved by the RIKEN Institutional Animal Care and Experimentation Committee, and all experiments were carried out in accordance with the approved guidelines.

### Animals

The netrin-G1^flox^ allele was generated by a gene-targeting strategy using MS12 ES cells derived from C57BL6 mice. We used the Red/ET recombination strategy to construct the targeting vector to delete exon 2 upon the expression of Cre protein. Exon 2 of netrin-G1 contains the start codon ATG and encodes the signal peptides and 25% of domain VI. We subcloned exon 2 and its flanking sequences from a BAC clone to a pDEST-diphtheria toxin A plasmid containing a diphtheria toxin A fragment cassette for negative selection in ES cells. A loxP-flanked PGK-gb2-neo cassette was inserted 5′ to exon 2 and the kanamycin/neomycin selection marker was further deleted by Cre recombination so that only a single loxP site remained. A neomycin-resistant gene cassette flanked by two Flp recombinase target sites and a single 3′ loxP site was inserted 3′ to exon 2. Sequencing was performed on the entire targeting vector and all the insertions and deletions were confirmed before ES cell electroporation. Targeted clones were identified by Southern blot analyses of *KpnI* digests with a 5′ flanking probe and *NcoI* digests with a 3′ flanking probe. Targeted ES cells were injected into C57BL/6J blastocysts to obtain chimeras. Male chimeras were mated with C57BL/6J females to obtain F1 mice, and the mutation heterozygotes were crossed with transgenic CAG-Flpe mice^16^ to excise the selection marker gene cassette. The heterozygous offsprings were further crossbred with C57BL/6J mice and interbred to obtain homozygous NetrinG1^f/f^ mice. The PCR primer pairs for genotyping were as follows:

g1-l-5: 5′-GACTGGTATCTCTAAGTTACCTAGGCTG-3′;

g1-l-3: 5′-CTTACATCCTTTTATAGCCCTCACATCTGC-3′;

g1-f-5: 5′-GAGAACGCTTGCTCAGGATCACTCCTTC-3′;

g1-f-3: 5′-CAGCCGTCCCAAAGTACACACTTAAAGGAG-3′;

cre-2: 5′-ACCTGATGGACATGTTCAGGGATCG-3′;

cre-3: 5′-TCCGGTTATTCAACTTGCACCATGC-3′.

The g1-l-5 and g1-l-3 amplified 282-bp and 369-bp bands for WT and NetrinG1-flox alleles, respectively. The g1-f-5 and g1-f-3 amplified 473-bp and 573-bp bands for WT and NetrinG1-flox alleles, respectively. The g1-l-5 and g1-f-3 amplified 470-bp bands for when netrin-G1 was deleted by Cre recombinase. The cre-2 and cre-3 amplified 108-bp bands for Cre recombinase.

Different strains of conditional KO mice were produced according to the following breeding strategy: NetrinG1^flox/flox^ × Cre line to obtain (NetrinG1^f/+^/Cre^+^) mice, then (NetrinG1^f/+^/Cre^+^) × NetrinG1^f/f^ to obtain (NetrinG1^f/f^: Cre^+^) experimental group mice and (NetrinG1^f/f^:Cre^−^) control group mice. As Emx1-Cre mice induce germ-line recombination at a low frequency, NetrinG1^f/−^:Cre^+^ and NetrinG1^f/−^:Cre^−^ mice were used as cKO and control mice. Only male mice (3–6 months of age) were used for further analysis.

The Emx1-Cre, Vgat-Cre, and 5HTT-Cre transgenic mouse lines were described previously^19^,^20^,^28^,^58^. The ZI-Cre line was an independent line generated with the vector for the Vgat-Cre line. To generate the Pkcd-Cre line, a BAC clone was used as described previously^20^. Among the multiple lines obtained, a line (P) with high selectivity of recombination in the dorsal thalamic nuclei was selected.

### Antibodies and immunohistochemistry

Primary antibodies used in this study included rabbit polyclonal antibodies for netrin-G1^12^, rabbit polyclonal anti-c-Fos (Ab-5, Calbiochem), rabbit polyclonal anti-GABA (Sigma), mouse monoclonal anti-NeuN (Chemicon), rabbit polyclonal anti-ß-galactosidase (MP Biomedicals), and mouse monoclonal anti-ß-galactosidase (Promega). The secondary antibodies used for immunofluorescence were Alexa Fluor 488 and 546 donkey anti-mouse or rabbit IgG (Molecular Probes). Biotinylated goat anti-rabbit IgG and a Vectastain ABC kit (Vector Laboratories) were used for c-Fos staining. Mice for c-Fos analysis were firstly exposed to EPM or FC training and then sacrificed and perfused 100 minutes later. The c-Fos staining was performed as described previously^59^. Rabbit polyclonal antibodies against Netrin-G1 were used as described previously^12^.

### Imaging and image quantification

Images of c-Fos staining were acquired with a Nanozoomer (Hamamatsu Photonics) and the regions of interest (ROIs) were manually selected according to the mouse brain atlas^60^. The sections were anatomically matched to the mouse brain atlas and the settings for image acquisition were kept constant. The number of c-Fos-positive neurons was counted using a customized Matlab program, SpotDetection. The program determined the number of c-Fos-positive neurons and the size of the mask area. The normalized value of each section = [number of c-Fos-positive neurons/mask area (pixel number)] × 10^5^, and the mean normalized value for each ROI was calculated. The analysis was performed blind to the tissue genotype. To quantify the colocalization of GABA and ß-gal, images were acquired using a FV-1000 Olympus confocal microscope. The ROIs were selected manually according to the mouse brain atlas^60^. The number of cells was counted manually using ImageJ. The colocalization was judged manually under the FV10-ASW3.0 Viewer (Olympus). 400x magnification images were used to analyze the cortex, zona incerta, thalamus and amygdala. 100x magnification images were used to analyze the hippocampus due to the sparse and limited number of ß-gal positive cells in the hippocampus.

### *In situ* hybridization

Preparation of the probes for *Netrin-G1* and digoxigenin-labeled antisense riboprobes, and hybridization of free-floating sections were performed according to previously described procedures^12^.

### Behavioral tests

The EPM is widely used to evaluate anxiety-related behaviors in rodents. This apparatus comprises two open arms (8 cm × 25 cm, no walls), two closed arms of the same size with 15-cm high walls, and a central platform (8 cm × 8 cm) at the intersection of the arms; the arms are positioned at 90-degree angles to each other with each arm type (open and closed) located directly across from each other. The maze was elevated 50 cm above the floor and illuminated at 200 lux. Each mouse was placed on the central platform of the maze, and exploratory behavior was monitored with a CCD camera for 10 min and analyzed with the NIH Image EP software (O’Hara & Co.). The parameters included the total distance traveled, the number of entries into each arm, and the amount of time spent in each arm and the central platform. The FC test was used to assess fear learning and memory in mice. On day 1, the animal was placed in a conditioning chamber. After 2 min free exploration, each mouse was exposed to two tone-footshock pairings (tone, 30 s, 80 db white noise; footshock, 2 s, 0.75 mA, co-terminating with the tone). One minute after the second footshock, the mouse was returned to its home cage. The contextual fear memory test was conducted on day 2. Each mouse was placed in the conditioning chamber for 5 min during which neither tone nor shock was presented. Conditioned fear memory to the tone was investigated on day 3 by placing each mouse in a novel chamber. After 2 min free exploration, each mouse was exposed to three tones (58 s, 80 db white noise, separated by a 2-s interval). Images were captured using a CCD camera and the area (in pixels) in which the mouse moved was measured. Freezing was defined as a lack of movement such that fewer than 45 pixels changed between successive video frames for at least 2 s, and was assessed and averaged over 1 min time bins. Freezing Percentage = (total freezing time/total testing time) × 100.

### Statistics

The data were analyzed using Student’s two-tailed t test and two-way ANOVA with IBM SPSS Statistics (ver. 21). All values are expressed as mean ± SEM. P values less than 0.05 were considered significant.

## Additional Information

**How to cite this article**: Zhang, Q. *et al*. Netrin-G1 regulates fear-like and anxiety-like behaviors in dissociable neural circuits. *Sci. Rep.* **6**, 28750; doi: 10.1038/srep28750 (2016).

## Supplementary Material

Supplementary Information
