# Supplementary material for: Netrin-G1 regulates fear-like and anxiety-like behaviors in dissociable neural circuits
Source: Sci Rep. 2016 Jun 27;6:28750. doi: 10.1038/srep28750 (PMC4921862; doi:10.1038/srep28750)
Supplement: Supplementary Information [file srep28750-s1.doc]

**Supplementary Figures**

**Netrin-G1 regulates fear-like and anxiety-like**

**behaviors in dissociable neural circuits**

Qi Zhang^1^, Chie Sano^1^, Akira Masuda^1^, Reiko Ando^1^, Mika Tanaka^1^, Shigeyoshi Itohara^1^,*

1. Laboratory for Behavioral Genetics, RIKEN Brain Science Institute, Wako, Saitama, 351-0198, Japan

*Corresponding author: Shigeyoshi Itohara, Laboratory for Behavioral Genetics, RIKEN Brain Science Institute, 2-1 Hirosawa, Wako, Saitama, 351-0198, Japan,

E-mail: [sitohara@brain.riken.jp](mailto:sitohara@brain.riken.jp)

Phone: +81-48-467-5156

Fax: +81-48-467-5180


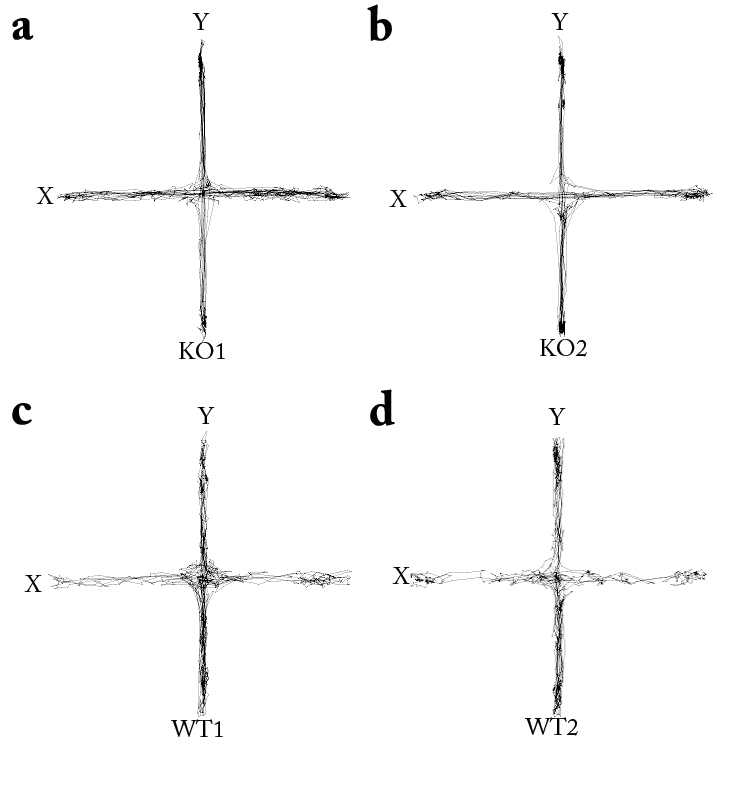


**Supplementary Fig. 1. Netrin-G1 KO mice showed decreased anxiety in EPM compared to WT mice.**

Video-tracking data illustrating that netrin-G1 KO mice (a,b) displayed no preference in exploring the closed arms (y axis) versus the open arms (x axis), contrary to WT mice (c,d) which showed a preference for the closed arms (y axis). Quantitative analysis using c-Fos labeling was conducted for on the KO mice and WT mice (see Fig xxx).


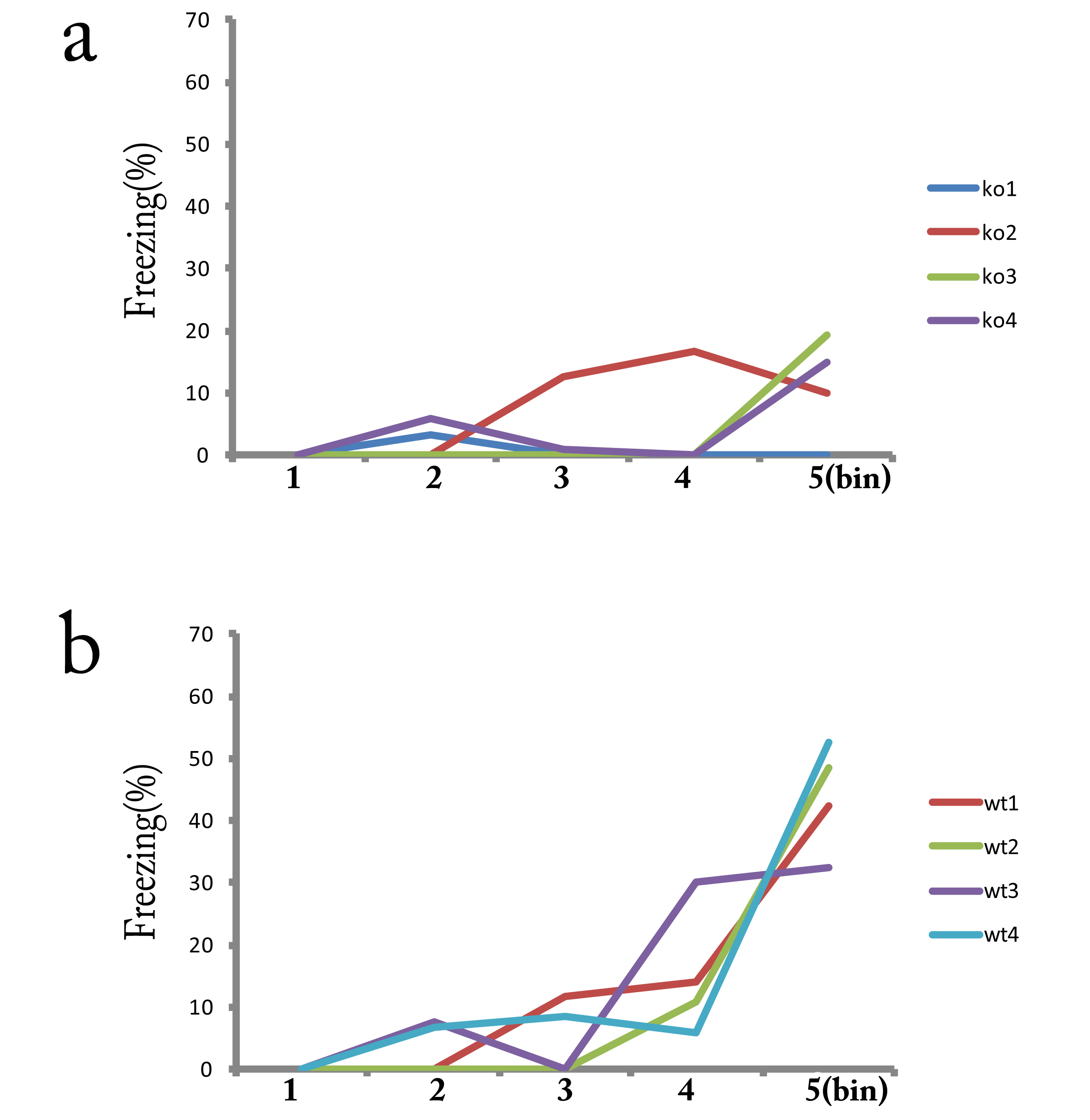


**Supplementary Fig. 2. In the conditioning phase of FC test, netrin-G1 KO mice showed an attenuated freezing response comparing to WT mice.**

(a) Freezing percentages of the four individual KO mice used for c-Fos quantitative analysis. (b) Freezing percentage of the four individual WT mice used for c-Fos quantitative analysis. Netrin-G1 KO mice displayed attenuated levels of freezing compared to WT mice.


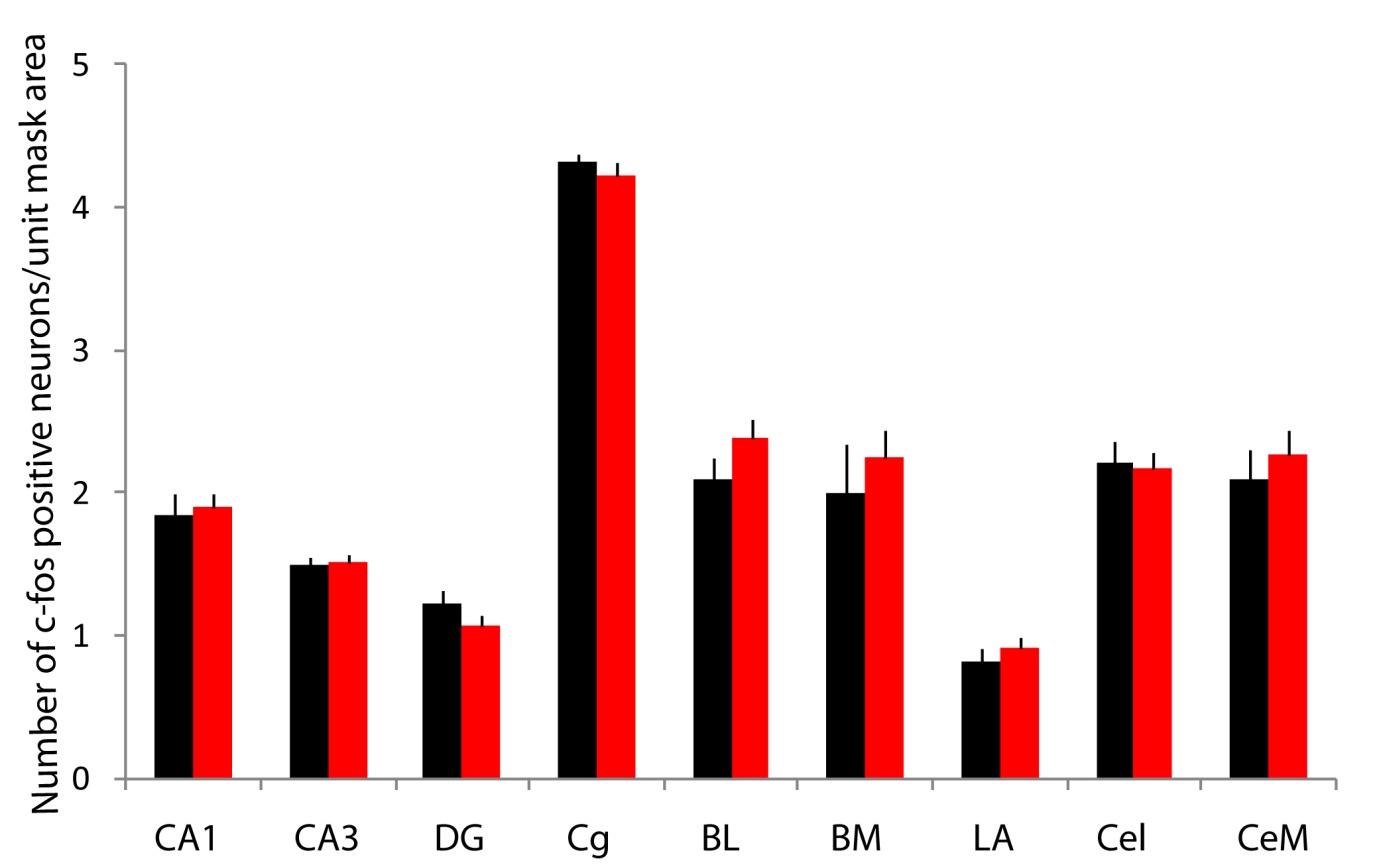


**Supplementary Fig. 3. Comparison of c-Fos expression pattern between home-caged netrin-G1 KO mice and WT control mice.**

The number of c-Fos positive cells of each ROI was compared between WT and netrin-G1 gKO mice with Student’s two-tailed t test (n=8, 4sections×2animals per genotype). The number of c-Fos positive cells was not significantly different for all the brain regions examined, including CA1 (p=0.74), CA3 (p=0.94), dentate gyrus (DG; p=0.24), cingulate cortex (Cg; p=0.33), basolateral amygdala (BL; p=0.20), basomedial amygdala (BM; p=0.53), lateral amygdala (LA; p=0.51), lateral central nucleus of the amygdala (CeL; p=0.88), and medial central nucleus of the amygdala (CeM; p=0.55). Black columns represent WT and red columns represent gKO, and data are represented as mean + SEM.


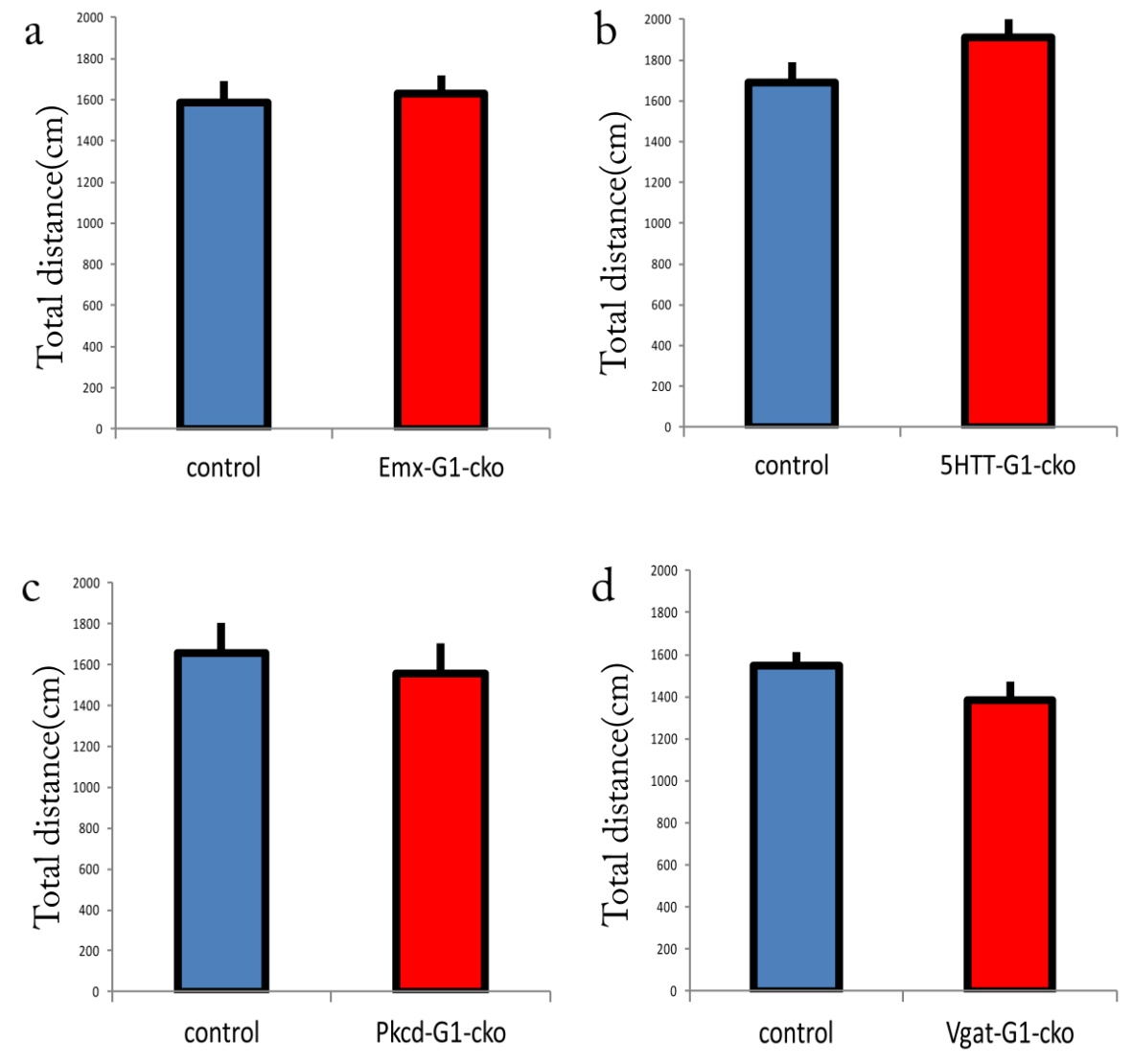


**Supplementary Fig. 4. Comparison of total distance travelled between different lines of netrin-G1 cKO mice and their littermate control mice.**

The total distances were compared with Student’s two-tailed t test. Total distance moved did not significantly differ for any of the groups. (a) Emx-G1-cKO and its control group (n=11/group: p=0.79). (b) 5HTT-G1-cKO and its control group (n=12/group: p=0.15). (c) Pkcd-G1-cKO and its control group (n=14/group: p=0.63). (d) Vgat-G1-cko and its control group (n=9/group: p=0.16). Blue columns represent WT and red columns represent cKO, and data are represented as the mean + SEM.


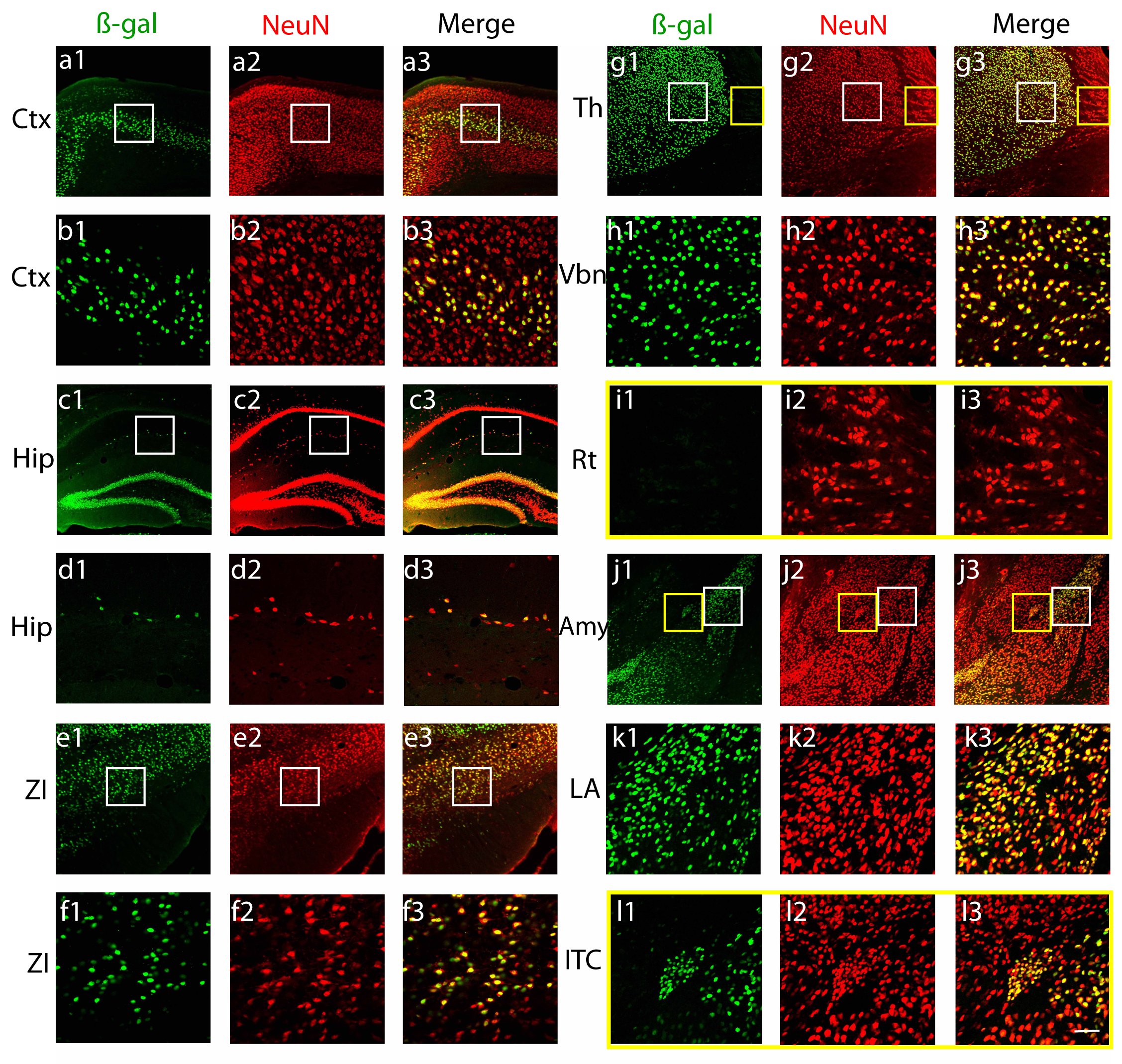


**Supplementary Fig. 5. Netrin-G1 expression in neurons.**

Coronal sections containing different brain regions were co-immunostained with antibodies against ß-gal (1: green) and neuN (2: red), and colocalization of the two signals was observed (3: green+red). (a1-a3) represent cortex (Ctx) and (b1-b3) are higher magnified images of the areas indicated by the white boxes in a1-a3. (c1-c3) represent hippocampus (Hip) and (d1-d3) are higher magnified images (white boxes in c1-c3). (e1-e3) represent zona incerta (Zi) and (f1-f3) are higher magnified images (white boxes in e1-e3). (g1-g3) represent thalamus and (h1-h3) and (i1-i3) are higher magnified images from Vbn (white boxes in g1-g3) and reticular nucleus (Rt; yellow boxes in g1-g3). (j1-j3) represent amygdala and (k1-k3) and (l1-l3) are higher magnified images from LA (white boxes in j1-j3) and the dorsal intercalated cluster (ITCd; yellow boxes in j1-j3). The higher magnification images show that all ß-gal positive cells are colocalized with neuN across all the brain regions tested. Scale bar: 120 µm (a, c, e, g, j); 30 µm (b, d, f, h, i, k, l).
